# There Is Little Difference in the Peak Movement Demands of Professional and Semi-Professional Rugby League Competition

**DOI:** 10.3389/fphys.2019.01285

**Published:** 2019-10-14

**Authors:** Rich D. Johnston, Paul Devlin, Jarrod A. Wade, Grant M. Duthie

**Affiliations:** ^1^School of Behavioural and Health Sciences, Australian Catholic University, Brisbane, QLD, Australia; ^2^Institute for Sport, Physical Activity and Leisure, Leeds Beckett University, Leeds, United Kingdom; ^3^Brisbane Broncos Rugby League Football Club, Brisbane, QLD, Australia; ^4^South Sydney Rabbitohs Rugby League Football Club, Sydney, NSW, Australia; ^5^School of Behavioural and Health Sciences, Australian Catholic University, Sydney, NSW, Australia

**Keywords:** microtechnology, activity profiles, rolling average, team sport, accelerometer, acceleration

## Abstract

Previous research has quantified the peak movement demands of elite rugby league match-play, but the peak accelerometer load or the semi-professional peak demands remain unknown. The aim of this research was to determine the peak movement demands of professional and semi-professional rugby league competition. Wearable microtechnology devices tracked the physical activity profiles of players during 26 professional (*n* = 351 files) and 22 semi-professional (*n* = 267 files) matches. Following each match, data were exported in raw form to extract the peak 1- to 10-min periods for speed, average acceleration, and accelerometer load of each player, using a rolling average method. To determine the difference between playing levels (professional vs. semi-professional) and position (forwards vs. backs), linear mixed models were used. The intercept and the slope were calculated based on the power law relationship to provide the peak, and rate of decay, of each dependent variable. Cohen’s effect size (ES) statistic was used to determine the magnitude of differences between positions and playing level. There was little difference between playing standards, with only *small* differences in running speed, with a greater intercept and slope for the professional forwards compared with semi-professional forwards (intercept ES: 0.37; 90%CL: 0.19 to 0.55; slope ES: 0.35; 0.15 to 0.55). For positional comparisons (forwards vs. backs), there was no difference in running speeds at the professional level, but there was substantially greater running speed for backs compared to forwards in semi-professional competition, with *small* to *moderate* differences (ES range: 0.60–0.39). Both professional and semi-professional forwards showed *small* to *moderately* higher accelerometer load compared to backs, which increased with period duration (ES range: 0.22–0.79). Similarly, acceleration demands were greater for forwards compared to backs across both playing standards, with *moderate* to *large* differences (ES range: 0.52–0.96). Overall, the results of this study show that there is a *small* difference in the peak running speed for forwards in professional competition, but otherwise there are no meaningful differences in movement demands of professional and semi-professional rugby league match-play. Forwards display greater acceleration and accelerometer load across a number of rolling average durations compared to backs.

## Introduction

Rugby league is a team sport, that is intermittent in nature, involving periods of high-intensity activity (e.g., collisions, accelerations) interspersed with periods of low-intensity activity (e.g., walking and jogging). Over the course of a game, players typically cover 85–100 m^.^min^–1^ depending on playing standard and position (Johnston et al., 2014a). However, the speeds during professional National Rugby League (NRL) and European Super League (ESL) competition can be as high as 172–154 m^.^min^–1^ during the peak passages of play (Delaney et al., 2015; Weaving et al., 2018). Whilst this information can be used to guide the intensities of training drills, there is no information regarding the peak periods of senior semi-professional competition. Previous work has shown greater average match demands in professional vs. semi-professional competition (McLellan and Lovell, 2013), but once again this does not provide information on the most demanding passages of play. In Australia, state-wide semi-professional competitions in New South Wales and Queensland provide the direct pathway to professional competition. Elite junior and contracted NRL players not selected for NRL will be expected to play in these competitions. As such, it is important to understand the demands of these competitions in comparison to NRL to guide prescription of training for these players.

Although the peak speeds highlight large increases in intensity above match average, they do not paint the whole picture regarding the locomotion demands of rugby league. The peak speeds previously documented, 172–154 m^.^min^–1^, could all be accumulated via low-speed activity. Indeed, over an 80-min game, players will only cover 300–500 m at high speeds (Johnston et al., 2014a). Given the intermittent, stop-start nature of match-play, other activities must be quantified when defining the peak movement demands of rugby league match-play. The 10-m rule, and passages of play where the ball is restricted to a small area of the field, means players are not presented with the opportunity to accumulate large distances at high speeds. Therefore, the ability to change momentum rapidly and repeatedly is vital, particularly for players in the center of the field (Delaney et al., 2016). The widely documented reliability and validity issues associated with assessing accelerations using microtechnology devices (Scott et al., 2016; Delaney et al., 2017), meant research has been somewhat limited when it comes to quantifying this likely important aspect of the game. However, the average acceleration and deceleration metric, which provides a mean of the magnitude of changes in speed over a given duration, has be shown to offer good reproducibility (Delaney et al., 2017; Thornton et al., 2018). This led to the quantification of the peak acceleration demands of professional NRL competition, with similar peak 1-min values for all positions (1.22–1.28 m⋅s^–2.^min^–1^), however, as the period duration increased, outside backs present with the lowest average acceleration demands (Delaney et al., 2016). This is unsurprising given their distal position to the center of the field, where they have more space to move in and time to react to the play. Since then, the peak movement (Weaving et al., 2018) and contact and movement (Johnston et al., 2019) demands of ESL and NRL have been defined. Despite this, the peak acceleration demands of semi-professional competition are yet to be quantified.

Whilst the acceleration profiles do offer practitioners a variable that may be more likely to encompass the intermittent nature of match-play and certain training drills compared to speed, it still does not truly account for the multidirectional nature of rugby league training and matches. The triaxial accelerometers housed within the microtechnology devices quantify gravitational forces across all three planes (mediolateral [x], vertical [y], and anteroposterior [z]) of movement, and therefore may provide a more global metric of the locomotive worked performed by players. Manufacturers of microtechnology devices have inbuilt metrics that sum accelerations in all three planes to provide a metric termed accelerometer load. Indeed, increasing the change of direction demands of running drills results in increases in accelerometer load, with the greatest increases coming in the mediolateral plane compared to straight-line running (Hulin et al., 2018). As such, these devices may be able to provide a more holistic measure of the multidirectional demands imposed on players in training and competition. Despite this, no study has assessed the peak accelerometer load of rugby league match-play.

The aim of this research was to determine the peak speed, acceleration and accelerometer load of matches across professional and semi-professional competitions. It was hypothesized that the peak match demands would be greater during professional compared to semi-professional competition. Forwards would display greater acceleration and accelerometer load profiles, yet lower speed profiles than backs.

## Materials and Methods

To test our hypothesis, physical activity profiles were tracked during professional NRL matches and semi-professional Queensland Intrust Super Cup (ISC) matches across the 2018 season using microtechnology devices. The data was then exported in its raw form in order to extract the peak 1- to 10-min periods for each player during each match.

### Subjects

Twenty-four professional (age = 25.4 ± 4.1 years; stature = 187.4 ± 6.4 cm; body mass = 100.4 ± 9.8 kg) and 26 semi-professional players (age = 25.6 ± 3.2 years; stature = 184.5 ± 7.4 cm; body mass = 98.7 ± 10.4 kg) took part in this study from two clubs playing in the NRL and ISC competitions, respectively. The study gained ethical approval prior to the start of the data collection. All data were collected as part of the routine operations of the club and were de-identified prior to analysis.

### Experimental Protocol

Microtechnology devices were used to track the physical activity profiles of players during 26 NRL (10 losses, 16 wins; *n* = 351 match files) and 22 ISC matches (10 losses, 12 wins; *n* = 267 match files). The microtechnology units used in this study comprised a 10 Hz multi-global navigation satellite system (GNSS) chip, a 100 Hz triaxial accelerometer, 100 Hz gyroscope and 10 Hz magnetometer (StatSports Apex, Newry, Northern Ireland). These units have shown acceptable validity and reliability for measuring distances and speeds common to team sports (Beato et al., 2018; Thornton et al., 2018). Each player was assigned a specific unit at the start of the season and this was maintained across all matches. Prior to the start of each match, the units were switched on approximately 20-min prior to the warm-up and fitted into a padded compartment sewn into the playing jersey. All players were measured for jerseys at the start of the season to ensure it was tight fitting, in order to minimize measurement noise, particularly when it comes to accelerometer load (McLean et al., 2018). During matches, information was fed to a laptop running Apex software (version 3.0.01191) connected to a beacon via an ultra-wideband secured wireless network. This allowed the sport scientist to synchronize the recording of data at the start and end of each half. The quality of the data was determined by recording the horizontal dilution of position (HDOP), and the number of satellites; any files with a HDOP >1.5 were removed from the analysis. Subsequently, two ISC files, and 15 NRL files were removed. For ISC games, there was an average HDOP of 0.45 ± 0.13 and 20.0 ± 1.6 satellites. For NRL games, there was an average HDOP of 0.76 ± 0.25 and 17.7 ± 1.9 satellites.

Following each match, the data files were downloaded using the manufacturer provided software and then exported in their raw form into a comma delimited file (csv), with each row representing a GNSS data point for each player. Once exported, moving averages were calculated over 1- to 10-min epochs for accelerometer load (Total Loading; AU⋅min^–1^), speed (m⋅min^–1^), and acceleration (m⋅s^–2.^min^–1^) in RStudio (Version 1.1.383, RStudio, Boston, MA). For GPS variables (speed, acceleration) sampled at 10 Hz, a minute rolling average included 600 data points, while for 100 Hz accelerometer load the 1-min rolling average included 6000 data points. Subsequently, the peak 1- to 10-min periods were extracted for each player for each match file. Accelerometer load, termed *Total Loading* in the Apex software, was calculated by summing the square root of change in gravitational forces in the mediolateral (x), vertical (y), and anteroposterior (z) planes using the manufacturers formula embedded within the Apex software; the formula is protected by a non-disclosure statement so cannot be presented in the paper. Speed was provided in the manufacturer supplied export, while average acceleration and deceleration was calculated as the absolute rate of change of speed ms^–2^.

### Statistical Analysis

Data were assessed for normal distribution using a Shapiro-Wilk test to determine whether parametric testing was appropriate. To determine the difference in speed, accelerometer load, and acceleration demands across 1- to 10-min periods linear mixed models were used. Separate models were built for each dependent variable across each duration, with match number and player identification number used as random effects and competition (NRL vs. ISC), position (forwards vs. backs) and match half (first vs. second) used as fixed effects. In addition, to determine the practical meaningfulness of any differences, Cohen’s effect size (ES) statistic and 90% confidence intervals (CI) were used, with thresholds of 0.00–0.19, *trivial*; 0.20–0.49, *small*; 0.50–0.79, *moderate*; and ≥0.80, *large* being used. A threshold likelihood of ≥75% of a small effect (0.20) was used to determine a substantial ES difference (Hopkins et al., 2009).

Additionally, to estimate intensities for each dependent variable over durations longer than 10 min, a power law relationship was used (Katz and Katz, 1994, 1999). This method describes the relationship between non-linear, but dependent relationships of two variables, in this case, microtechnology device output, and time (1- to 10-min) to give the equation:

$$c={\mathrm{yx}}^{n}$$

where n and c are constants.

A plot of log (x) against log (y) provides a straight line with intercept (y) and slope (n.) Linear regressions provided the slope and intercept for each variable (speed, accelerometer load, and average acceleration) from each match file. In the present study, where c is intensity, y is intercept, x is time, and n is slope:

$$\mathrm{Intensity}=\mathrm{intercept}\times \left({\mathrm{time}}^{\mathrm{slope}}\right)$$

**FIGURE 1:**
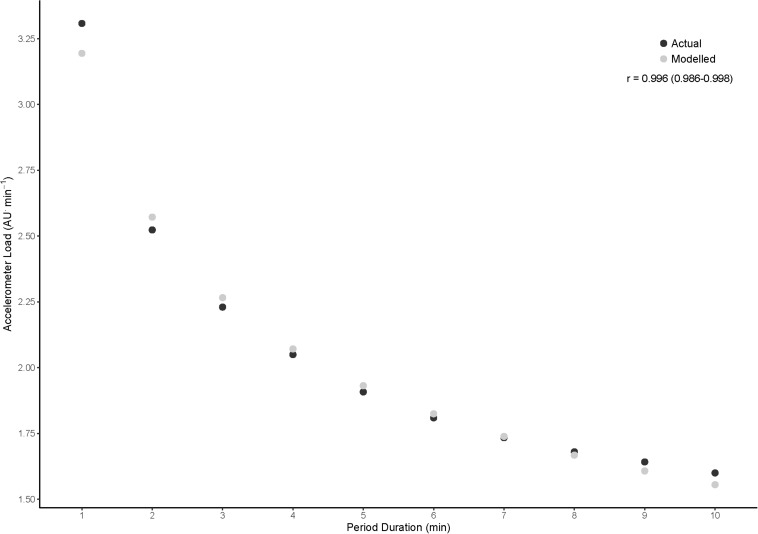
A comparison of peak accelerometer load for backs from actual and modeled data taken from the power law analysis and Pearson’s correlation between the two data sets.

This equation provides a measure of intensity based on speed, accelerometer load, or acceleration for a given time period. An example of this can be seen in Figure 1, with actual and modeled data being presented for accelerometer load in the backs.

## Results

### Between Playing Levels

As shown in Table 1, there was greater running speed (*small*) intercept for the NRL forwards compared with ISC forwards (ES: 0.37; 90%CL: 0.19 to 0.55), with *small* differences over the 1- (0.39; 0.21 to 0.57) and 2-min (0.25; 0.07 to 0.43), periods. Along with greater peak speed, NRL forwards also showed a greater (*small*) slope for running speed (0.35; 0.15 to 0.55). There were no differences in accelerometer load intercept or slope and average acceleration between forwards across playing levels.

**TABLE 1:** Intercept and slope values for estimating intensity by duration for relative distance, accelerometer load, and average acceleration across NRL and ISC competitions.

	**NRL forwards**	**ISC forwards**	**NRL backs**	**ISC backs**
**Relative distance**				
Intercept (m^.^min^–1^)	167.2 ± 23.4	161.1 ± 13.1^∗^†	168.0 ± 32.0	167.5 ± 13.4
Slope	−0.24 ± 0.07	−0.22 ± 0.03^∗^	−0.24 ± 0.06	−0.22 ± 0.04
**Accelerometer load**				
Intercept (AU^.^min^–1^)	3.26 ± 0.47	3.31 ± 0.38	3.16 ± 0.42	3.25 ± 0.47
Slope	−0.28 ± 0.05†	−0.28 ± 0.05†	−0.31 ± 0.07	−0.31 ± 0.06
**Average acceleration**				
Intercept (m^.^s^–2^)	1.35 ± 0.12†	1.36 ± 0.09†	1.28 ± 0.09	1.28 ± 0.08
Slope	−0.18 ± 0.05	−0.18 ± 0.03	−0.18 ± 0.03	−0.18 ± 0.03

*Data are presented as means ± standard deviation. NRL = National Rugby League (professional); ISC = Intrust Super Cup (semi-professional). ^∗^ denotes a significant difference (*p* < 0.05) between competitions; † denotes a significant difference between playing positions from the same competition (e.g., NRL backs vs. NRL forwards).*

There were no differences between backs across NRL and ISC competitions (Table 1). With only *small*, *unclear* differences in higher running speeds across periods 3- to 7-min in ISC backs compared to NRL backs (ES range: 0.20–0.24). Similarly, for accelerometer load, there were no clear differences other than a *small*, higher load in the 2- and 3-min periods. No differences were seen across all durations for average acceleration.

### Position Comparison

**FIGURE 2:**
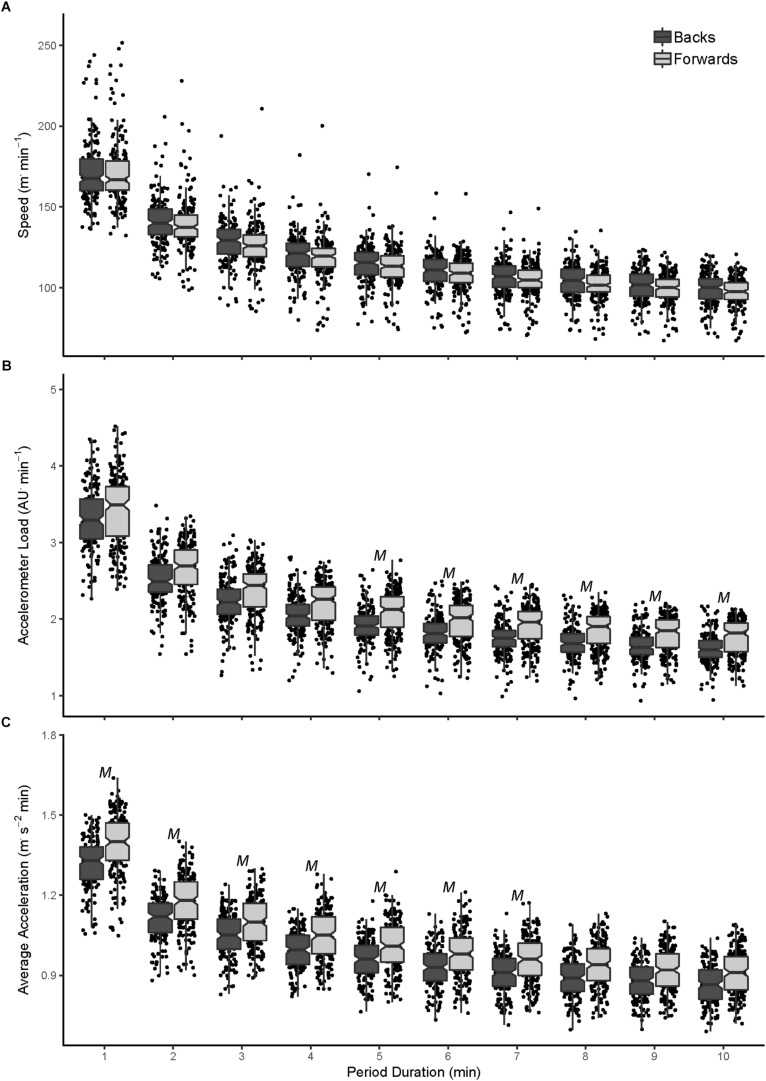
Peak speed **(A)**, accelerometer load **(B)**, and average acceleration **(C)** profiles for professional forwards and backs over 1- to 10-min moving average periods. *M*, moderate effect size difference (0.50–0.79) between forwards and backs.

In the NRL, there was no difference in running speed intercept or slope between forwards and backs (Table 1), with only *trivial* differences across each period (Figure 2A). There was no difference in intercept for accelerometer load for between positions, but the slope was *moderately* lower (0.53; 0.43 to 0.71) for forwards. This resulted in *small* to *moderately* higher accelerometer load for forwards, which became greater as the duration increased (ES range: 0.22–0.67; Figure 2B). There were greater acceleration demands in forwards (0.66; 0.44 to 0.88), but no difference in the slope between positions (0.08; −0.59 to 0.75). The difference in acceleration demands became progressively smaller, with *moderately* greater profiles over 1- to 7-min periods (ES range: 0.62–0.52; Figure 2C).

**FIGURE 3:**
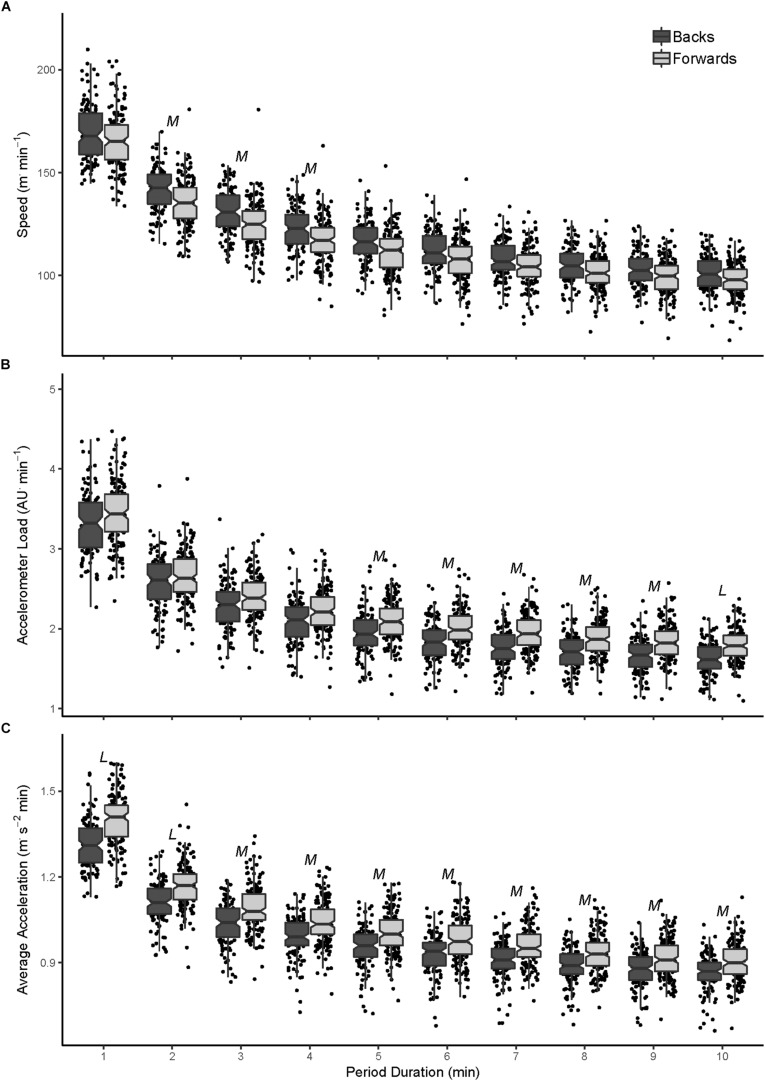
Peak speed **(A)**, accelerometer load **(B)**, and average acceleration **(C)** profiles for semi-professional forwards and backs over 1- to 10-min moving average periods. *M*, moderate effect size difference (0.50–0.79); *L*, large effect size difference (>0.79) between forwards and backs.

In the ISC competition, backs had a greater speed intercept than forwards (0.49; 0.10 to 0.88) with no difference in the slope. There was greater running speed across 2- to 9-min periods for backs compared to forwards, with *small* to *moderate* differences that tended to decrease with period duration (ES range: 0.60–0.39; Figure 3A). In contrast, forwards showed a lower slope (0.68; 0.44 to 0.92) for accelerometer load, with *small* to *moderately* greater load during 3- to 10-min periods that became larger as the period duration increased (ES range: 0.41–0.79; Figure 3B). There were also greater acceleration profiles in forwards (Figure 3C), with a *large* difference in intercept for forwards (0.95;0.64 to 1.26), and *large* to *moderate* differences across 1- to 10-min periods (ES range: 0.96–0.62), these differences became smaller as the period duration increased.

## Discussion

This is the first study to compare the peak match demands of professional and semi-professional rugby league players, and the first to quantify the peak accelerometer loads of rugby league match-play across any standard. Contrary to our hypothesis, this study found that there was no difference in the peak demands for backs between playing standard, and only small differences in peak speed for forwards. Across both playing standards, forwards had greater acceleration and accelerometer profiles across peak periods compared with backs; the differences in accelerometer became greater as the period duration increased. In comparison, the difference in the acceleration demands between positional groups decreased as duration increased. This study highlights that the peak demands of professional and semi-professional competition are similar. Whilst the peak speed demands may be higher for backs, forwards are exposed to greater acceleration and accelerometer load demands. Coaches can use this information to track the intensity of training drills relative to the speed of match-play. Further, training drills for positional groups should reflect the differences in the peak demands of match-play observed.

There was little difference in the peak demands of match-play across professional and semi-professional competition, other than the *small* greater peak speed of professional match-play for forwards, yet a greater slope, indicating faster declines in running speed in professional forwards over the peak periods. Previous research has highlighted greater match demands in professional compared with the semi-professional state competitions (Sirotic et al., 2009; McLellan and Lovell, 2013). However, these studies were several years ago, and changes to competition structure in this time, and increased professionalization of sub-elite competition may have led to a narrowing of match intensity between playing standards. In addition, these studies only assessed the average match demands. The greater slope in professional forwards suggests that it is only over shorter periods that running speed is greater, and they are unable to maintain output over longer periods. Whilst the locomotor demands are similar, the concomitant contact demands may be greater for professional players (Gabbett, 2013; Johnston et al., 2014a; Hausler et al., 2016). Previous research has highlighted the significant energetic cost of physical contact in rugby league players (Norris et al., 2016; Costello et al., 2018), causing reductions in running intensity (Johnston et al., 2014c, 2015, 2016), and increases in markers of fatigue (Johnston et al., 2014b). A recent study has looked at the peak running and contact demands of professional competition (Weaving et al., 2018), but no studies have assessed the peak contact and running demands of semi-professional competition. Although peak demands may be similar, the activity prior to, and following these peaks, may be different and warrants future investigation. Given the lower peak speed for semi-professional forwards, players that are contracted to professional clubs, may need to be exposed to “top-up” work in-season, so, if selected, they are prepared for the additional running demands of professional competition.

The peak speed demands of professional competition reported in this study were greater than a previous study (Delaney et al., 2015), but similar to another (Delaney et al., 2016). In addition, the acceleration demands were greater in the present study compared to those previously (Delaney et al., 2016). This difference may be attributable to the physical characteristics of the players (Duthie et al., 2017), or reflective of the different microtechnology devices used between studies, even though raw data were exported (Thornton et al., 2018). Similarly to previous research, we once again showed there is a positional difference in the peak demands of competition at both professional and semi-professional levels. In semi-professional players, the backs had greater peak running speeds, with moderate differences during 2-, 3- and 4-min periods. This is likely a result of these players being located further from the center of play, where they have more room to move and reach higher velocities compared to forwards (Johnston et al., 2014a; Hulin et al., 2015). These differences, however, were only seen over shorter periods, which once again is likely reflective of their role on the field. They are less likely to have prolonged periods, where they are consistently involved in play, rather short bouts of high-speed activity such as supporting a line-break or during a kick chase. Indeed, following the most intense passages of play, backs show greater reductions in running intensity than forwards (Hulin et al., 2015). Unlike semi-professional players, there was no difference in the peak match speeds between forwards and backs in professional players. This may be reflective of the higher standard of competition, with superior technical and physical abilities (Gabbett et al., 2011a, b). In professional rugby league competition, forwards perform similar running intensities over peak match periods, in comparison with the backs, and training should reflect this. In semi-professional players, however, during training drills, particularly those over shorter periods (e.g., 1- to 5-min), backs should be covering greater distances than the forwards that include high-speed kick chases and line-breaks in-line with their positional roles.

Despite the greater, or similar peak speeds between forwards and backs in semi-professional and professional players respectively, there were greater acceleration and accelerometer loads for forwards across both playing levels. This is somewhat in accordance with previous work on the acceleration demands of rugby league (Delaney et al., 2016), where they highlighted the hooker and edge forwards had the greatest acceleration demands. There was no further breakdown of positions in the current study, so we cannot confirm which positions accounted for the increased demands in the forwards. These differences are likely reflective of the demands of the game, where forwards are located close to the center of the field; there is less space to reach high velocities, but more changes in velocity. For example, during a defensive set, forwards accelerate off the defensive line, fill space on the inside of the ball, and are involved in frequent collisions. This results in large and, or rapid changes in speed from either low, or standing starts and a greater number of directional changes and accelerations, which increases accelerometer and acceleration profiles (Hulin et al., 2018). Furthermore, it suggests that these metrics may be more reflective of the type of activities performed by forwards, which include short accelerative and decelerative actions, directional changes and repeated collisions (Gabbett et al., 2012; Hausler et al., 2016). Accelerometer load, is the sum of gravitational forces in three planes and therefore is likely a good holistic measure of load, although it may not adequately quantify the contact demands of competition (Hulin et al., 2018). Although this study suggests there is a disconnect between the peak speed periods and peak acceleration and accelerometer profiles, future work should look to ascertain this. Practitioners should look to utilize accelerometer and average acceleration metrics to quantify intensities of training drills, particularly those in confined spaces, that may be more important for forwards.

This study was the first to document the peak movement demands of semi-professional competition, and the first to quantify the peak accelerometer loads of any level of rugby league. In addition, whilst there was only a small difference between standards, with greater peak speeds for forwards, there were substantial differences between positional groups. Forwards across both playing standards showed greater acceleration and accelerometer load profiles compared with backs. This suggests there is a disconnect between peak speed and average acceleration and accelerometer load metrics. As such, they are likely to be useful for quantifying external workloads for rugby league players. Further, we also modeled the peak and decay of GNSS metrics in players across both standards of competition to allow practitioners to estimate the intensity of drills of varying durations with a high degree of confidence. Despite these findings, the data were collected from only one semi-professional and one professional club and therefore may be reflective of all players. Further, only the movement demands of competition were assessed, with no contact component included.

### Practical Applications

The peak intensities of rugby league match-play are below the intensities of typically prescribed conditioning drills. As such, this information should be used to guide the intensity of game-based technical-tactical training, where players are challenged to execute skills and decisions under match-like fatigue. Semi-professional forwards may require “top-up” drills in training over 1- to 2-min periods to be ready for professional matches. Drills at, or above those observed during 1- (173 m^.^min^–1^) and 2-min (138 m^.^min^–1^) peaks in professional competition should be used. Forwards should have more drills that are focused on accelerations and accelerometer load, particularly over the longer durations (5- to 10-min) compared with backs, these may come in the form of drills in more confined areas. Backs require more drills focused on open space where they can reach high speeds and cover high-speed distance such as kick chase to a line-break. Average acceleration and accelerometer load are useful variables to monitor the demands of training and competition as they appear to better reflect the intermittent activity and positional differences observed. The slope and intercept values that appear in Table 1 can be used to estimate the target intensity of a training drill using the equation Intensity = intercept × (time^slope^). Once practitioners know the length of their drill, this is raised to the power of the slope and then multiplied by the intercept. For example, a 5-min drill at 100% of the peak game intensity for accelerometer load would be calculated as 3.26 × (5^–0.28^) for forwards and 3.16 × (5^–0.31^) for backs. This would return target intensities of 2.07 AU^.^min^–1^ for forwards and 1.91 AU^.^min^–1^ for backs over the 5-min drill.

## Data availability statement

The datasets for this study will not be made publicly available because ethical approval was not granted by the ethics committee for the data to be shared. Only the publication of aggregated data was approved.

## Ethics Statement

All the data used in this study was collected as part of the clubs’ normal training procedures. The club provided approval for the researchers to use the data, with all data being de-identified prior to use. As such, informed consent was not required. This was approved by the Australian Catholic University Human Ethics Review Committee (approval number 2017-226R).

## Author Contributions

RJ was the main investigator. JW and PD were involved in the data collection and wrote the manuscript. GD was involved in the analysis, statistics, and wrote the manuscript.

## Conflict of Interest

The authors declare that the research was conducted in the absence of any commercial or financial relationships that could be construed as a potential conflict of interest.
